# Diversity, Distribution, and Host Blood Meals of Black Flies (Diptera: Simuliidae) in Laos

**DOI:** 10.3390/insects16101053

**Published:** 2025-10-16

**Authors:** San Namtaku, Wannachai Wannasingha, Waraporn Jumpato, Khamla Inkhavilay, Bhuvadol Gomontean, Komgrit Wongpakam, Chavanut Jaroenchaiwattanachote, Isara Thanee, Ronnalit Mintara, Peter H. Adler, Pairot Pramual

**Affiliations:** 1 Department of Science and Mathematics, Faculty of Science and Health Technology, Kalasin University, Kalasin 46230, Thailand; san.na@ksu.ac.th; 2 Center of Excellence in Biodiversity Research, Mahasarakham University, Mahasarakham 44150, Thailand; wannachai.w@msu.ac.th (W.W.); chavanut.j@msu.ac.th (C.J.); 3 Department of Biology, Faculty of Science, Mahasarakham University, Mahasarakham 44150, Thailand; waraporn.a2536@gmail.com (W.J.); bhuvadol.g@msu.ac.th (B.G.); isara.th@msu.ac.th (I.T.); ronnalitmintara@gmail.com (R.M.); 4 Center of Excellence in Biodiversity, National University of Laos, Vientiane 7322, Laos; khamla.inkhavilay@nuol.edu.la; 5 Walai Rukhavej Botanical Research Institute, Mahasarakham University, Mahasarakham 44150, Thailand; komwongpa@gmail.com; 6 Department of Plant and Environmental Sciences, Clemson University, Clemson, SC 29634, USA; padler@clemson.edu

**Keywords:** hematophagous insects, *Simulium*, vectors, pests

## Abstract

Adult black flies were collected from diverse habitats in five provinces of Laos. In total, 4659 adults (4570 females and 89 males) were collected. Morphological identification, supplemented with the mitochondrial cytochrome c oxidase I gene, revealed 12 species. The most abundant taxa were *Simulium khelangense*/*S. chumpornense* and *S. asakoae*/*S. myanmarense* with 58% (2628 of 4476) and 38% (1720 of 4476) of the total specimens, respectively. *Simulium asakoae*/*S. myanmarense* was the most geographically widespread taxon (65% of sites) from 174 to 1219 m above sea level. The remaining species were far less abundant (<2%) and found in only one or a few locations. Molecular identifications of vertebrate blood meals indicated that most hosts (57 of 66) were chickens. Five taxa, *S. asakoae*, *S. khelangense*, *S. myanmarense*, *S. aureohirtum*, and *S. striatum* group, fed on chickens, representing the first hosts records for the latter three taxa. *Simulium daoense* fed on water buffalo, marking the first record of this species in Laos and the first report of its blood hosts. The abundance and frequent use of domestic chickens as hosts suggests that *S. khelangense* and *S. asakoae* could be significant pests and might serve as vectors of avian blood parasites in Laos.

## Simple Summary

Black flies are small, blood-sucking insects, many of which are pests and vectors that transmit diverse pathogens and parasites to humans and other animals. Knowledge of species diversity, abundance, distribution, and biting habits are of fundamental significance for understanding their roles as pests and vectors. However, this information is limited in many countries, including Laos, where extensive research on black flies has recently begun. Here, we report the diversity, distribution, abundance, and vertebrate blood meals of black flies collected in five provinces (Vientiane, Bolikhamxai, Xaisomboun, Xiang Khouang, and Huaphan) of Laos. In total, 4659 specimens were collected and identified morphologically and with DNA barcoding; 12 species were found. *Simulium khelangense*/*S. chumpornense* and *S. asakoae*/*S. myanmarense* were the most abundant and geographically widespread black flies. Molecular identification of the host blood meals from nine species with blood-fed specimens revealed four hosts: chickens (*Gallus gallus*), turkeys (*Meleagris gallopavo*), water buffalos (*Bubalus bubalis*), and humans. This information is critical for monitoring the role of black flies as pests and vectors that might transmit pathogens and parasites to humans and other animals, such as economically important chickens.

## 1. Introduction

About 10–20% of the 2415 species of black flies (Diptera: Simuliidae) in the world are pests, and many transmit pathogens that affect the welfare of humans and domesticated animals [1]. The most significant simuliid-borne disease is human onchocerciasis or river blindness, caused by the filarial nematode *Onchocerca volvulus*, which is transmitted by at least 26 species of black flies [1]. Nearly 250 million people in affected areas require preventive treatment, and about 14.6 million people are infected [2]. Black flies also transmit other filarial nematodes, such as the causal agent (*Mansonella ozzardi*) of mansonellosis in humans, *Dirofilaria ursi* in bears, and at least 11 *Onchocerca* species of domestic and wild animals [1,3]. Black flies are also vectors of avian blood protozoa in the genera *Leucocytozoon* and *Trypanosoma*, which cause diseases in poultry [4].

The successful transmission of pathogens from one host to another depends on the probability of encounters between a vector and a susceptible host. Key factors in understanding disease epidemiology include the distribution, abundance, and biting behavior of the vectors [5,6]. This knowledge can be used to predict risk areas where the vectors and potential disease outbreaks might occur [7]. This information, however, is limited in tropical Asia even though the region harbors nearly 30% (719 of 2415) of the world’s black flies [8,9]. The distribution and abundance of adult black flies in tropical Asia have thus far been reported only in Thailand [10,11], Malaysia [12], and India [13]. The availability of suitable immature habitats, habitat types, and elevation are important factors related to adult distributions [10,11,14].

Biting behavior is one of the least-explored areas of black fly biology. Approximately 90% of all species require a blood meal for egg maturation [1]. In the Oriental Region, biting habits are known for only 12 species [11,15,16]. Among these, *Simulium asakoae* Takaoka and Davies, *S. nigrogilvum* Summers, and *S. nodosum* Puri are vectors of zoonotic filariae [9,15], and *S. chumpornense* Takaoka and Kuvangkadilok, *S. khelangense* Takaoka, Srisuka and Saeung, and *S. asakoae* are vectors of avian blood protozoa (*Leucocytozoon* and *Trypanosoma*) [17,18,19].

The first report of black flies (*S. fenestratum* Edwards) in Laos was more than 13 years ago [20]. Five years later, a new species, *S. laosense* Takaoka, Srisuka and Saeung, was described from the country [21]. In the last few years, explorations of the biodiversity and bionomics (e.g., vectorial roles) of black flies in Laos have been undertaken [22,23,24]. In total, 31 species in three subgenera (*Gomphostilbia*, *Nevermannia*, and *Simulium*) of the genus *Simulium* are now known in Laos [24], including potential vectors of *Onchocerca* nematodes [25,26]. *Simulium khelangense* in Laos is also a potential vector of these parasites [19]. Biting habits have been recorded for two species in Laos, *S. chamlongi* and *S. luculentum*, which feed on humans and water buffalos, respectively [16].

Our objective was to examine the diversity, distribution, and abundance of adult black flies in Laos and to identify the vertebrate hosts using a molecular approach. We aimed to identify candidate pests and vectors that warrant further investigation.

## 2. Materials and Methods

### 2.1. Collection and Identification

**Figure 1:**
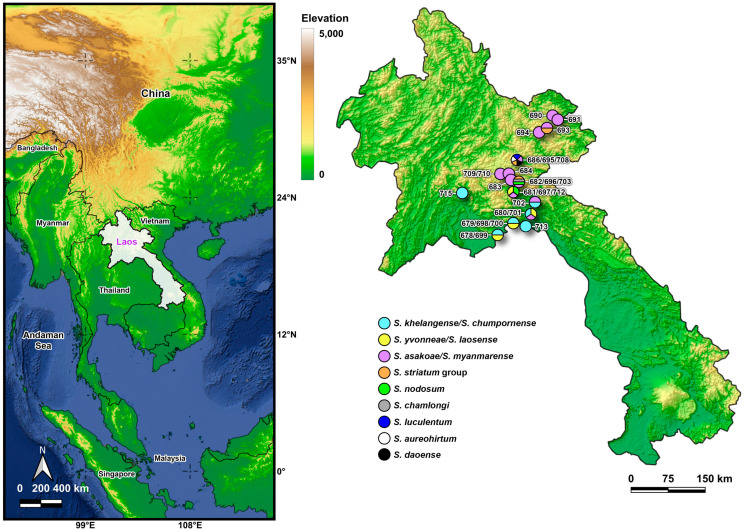
Map of collection sites of adult black flies in Laos. Colors represent species found at each sampling site.

In total, 30 collections were made from 20 sampling sites in five provinces (Vientiane, Bolikhamxai, Xaisomboun, Xiang Khouang, and Huaphan) of Laos between January and March 2025 (Table 1, Figure 1). Adult black flies were collected by sweeping an aerial net in a figure-eight motion 0.5–2.0 m above ground. Flies were fixed in 80% ethanol and stored at −20 °C until examination. Species were identified morphologically using available keys for Thailand [27] and Vietnam [28]. We complemented morphological identifications with DNA barcodes based on the mitochondrial cytochrome c oxidase I (COI) gene.

### 2.2. DNA Barcoding

Genomic DNA was extracted from entire specimens, using the GF-1 Nucleic Acid DNA Extraction Kit (Vivantis Technologies Sdn. Bhd., Subang Jaya, Malaysia). For DNA barcoding, the primers LCO1490 and HCO2198 [29] were used to amplify a fragment of approximately 650 bp of the COI gene, with PCR reaction conditions described by Tangkawanit et al. [30]. PCR products were checked using 1% agarose gel electrophoresis. Successful amplifications were purified using the PureDireX PCR CleanUp & Gel Extraction Kit (Bio-Helix, Taiwan, China). Purified PCR products were sequenced at the ATCG Company Limited (Thailand Science Park, Pathum Thani, Thailand) using the same primers as in PCR.

### 2.3. Molecular Identification of Vertebrate Blood Meals

Female flies were screened for the presence of vertebrate blood under a stereomicroscope, and only blood-engorged individuals were used for molecular analysis. Genomic DNA was extracted using the same method as for the DNA barcoding. The primers L14841 and H15149 [31], which specifically amplified the vertebrate cytochrome b (cyt b) gene (approximately 350 bp fragment), were used. The PCR reaction conditions for amplification of the vertebrate cyt b gene followed those of Malmqvist et al. [32]. PCR purification and sequencing were as described in the DNA barcoding study except for the primers used for sequencing cyt b.

### 2.4. Data Analysis

COI sequences (*n* = 103) generated in our study were deposited in the NCBI GenBank database under accession nos. PX314873–PX314957 and PX349369–PX349386. To use this marker for identification, the COI sequences of putative conspecifics in GenBank were retrieved and compared with sequences obtained from Laos. Phylogenetic analyses based on the neighbor joining (NJ) and maximum likelihood (ML) methods were used to examine genetic relationships of specimens from Laos and neighboring countries. The NJ analysis was performed in MEGA X ver. 10.1.8 [33] using the Kimura 2-parameter (K2P) model. Branch support was calculated using the bootstrapping method with 1000 replications. The ML tree analysis was conducted in MEGA X ver. 10.1.8 based on a general time-reversible (GTR) model with gamma distribution + invariant sites (G + I). The bootstrap method (1000 replications) was used to test branch support.

## 3. Results

### 3.1. Adult Black Fly Diversity and Distribution

**Figure 2:**
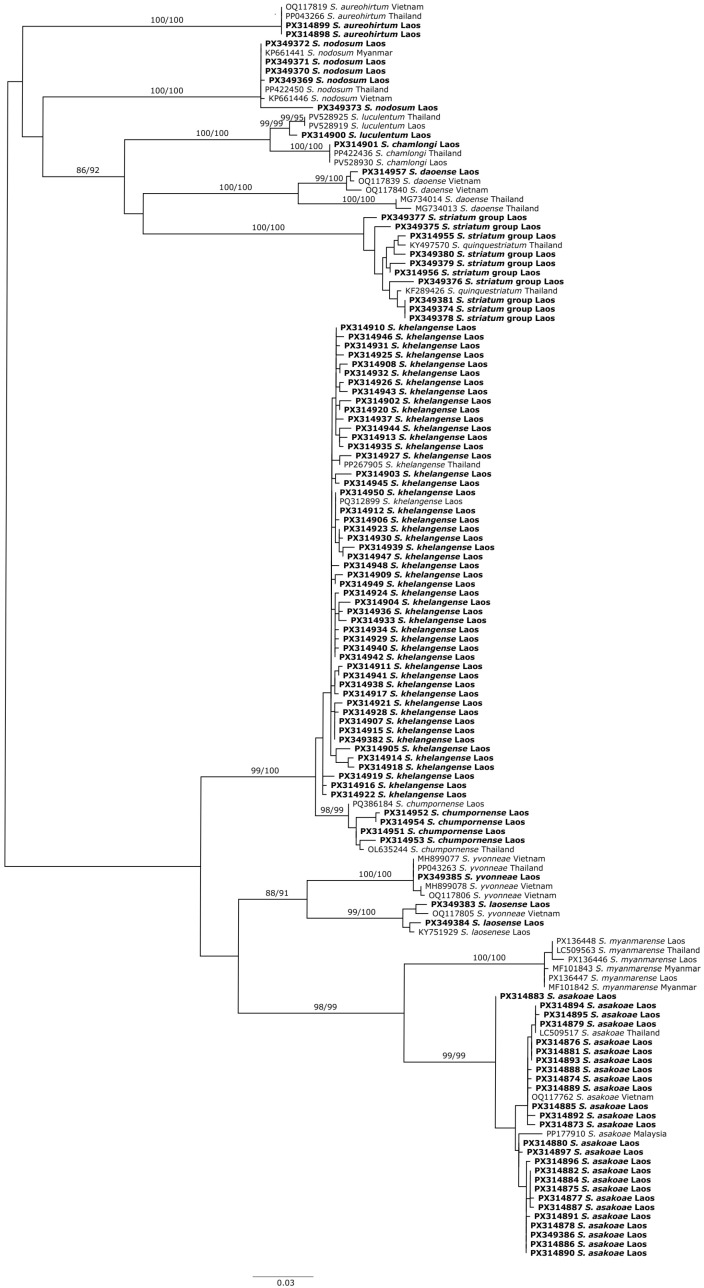
Maximum likelihood tree of adult black flies from Laos (bold) and conspecific COI sequences retrieved from the NCBI GenBank. Bootstrap values (1000 replicates) for ML and NJ analyses are above the branches.

We collected 4659 adult black flies (89 males and 4570 females, including 93 blood-fed individuals) (Table 1). Morphological identifications revealed eight species (*S. asakoae*, *S. khelangense*, *S. yvonneae*, *S. aureohirtum*, *S. chamlongi*, *S. luculentum*, *S. nodosum*, and *S. daoense*). Adults of the *S. striatum* species group are nearly isomorphic; therefore, we treated them as the *S. striatum* group. Phylogenetic analyses based on COI sequences of representative specimens that we collected from Laos, plus records of conspecifics in GenBank, showed that all species were monophyletic with strong (>99%) bootstrap support (Figure 2), with the exception of two individuals morphologically identified as *S. yvonneae*, which were part of a clade with *S. laosense* from the type locality [21]. We therefore treated these taxa as “*S. yvonneae*/*S. laosense*”.

Three blood-engorged females used for molecular identification of host blood sources were *S. myanmarense*, as reported by Adler et al. [24]. However, this species is morphologically similar to *S. asakoae* and might have been misidentified by Adler et al. [24]. Thus, we use the name “*S. asakoae*/*S. myanmarense*” to represent these taxa. Two distinct clades of *S. daoense* were revealed, both with strong support (>99%). A single specimen from Laos in our study belonged to the clade of *S. daoense* from the type locality in Vietnam [34], whereas specimens from Thailand formed another clade, suggesting that either flies from one of the clades were misidentified or cryptic species were involved. We refer collectively to two other species as *S. khelangense*/*S. chumpornense* because they are morphologically nearly indistinguishable and were identified only as *S. khelangense*. DNA barcoding of flies with blood meals, however, revealed that some specimens of *S. chumpornense* were present (Table 2). Overall, based on morphology and DNA, we found 12 species (Table 2).

Most specimens (96%) were *S. khelangense*/*S. chumpornense* (2725 or 58%) and *S. asakoae*/*S. myanmarense* (1788 or 38%). *Simulium asakoae*/*S. myanmarense* was geographically the most widespread, having been collected from 13 locations in the five surveyed provinces from 174 m to 1219 m above sea level (asl) (Table 2). *Simulium khelangense*/*S. chumpornense* was relatively more abundant than other species and was collected from 10 locations in two adjacent provinces (Bolikhamxai and Vientiane) at elevations below 500 m asl. The *S. striatum* group was found at three collection sites, two in Xiang Khouang Province and one in Huaphan Province, from 456 to 1219 m asl. *Simulium yvonneae*/*S. laosense* was collected from three sampling sites in Bolikhamxai Province between 162 m and 321 m asl. *Simulium nodosum* was collected from two sites, one at 321 m asl in Xaisomboun Province and the other at 995 m asl in Xiang Khouang Province. *Simulium aureohirtum*, *S. chamlongi*, and *S. luculentum* were each collected from a single site (Table 2).

### 3.2. Blood Meal Identifications

Morphological identifications of the 93 blood-engorged flies revealed seven taxa (*S. asakoae*, *S. aureohirtum*, *S. khelangense*, *S. chamlongi*, *S. luculentum*, *S. striatum* group, and *S. daoense*). When we used DNA barcoding, we found two additional species, *S. myanmarense* and *S. chumpornense* within *S. asakoae* and *S. khelangense*, respectively. Most blood-engorged females were *S. khelangense* (48 of 89, 54%) and *S. asakoae* (27 of 89, 30%). Among 93 blood-engorged females, 66 (71%) were successfully sequenced for the vertebrate cyt b gene, revealing four host sources: chickens (*Gallus gallus*), turkeys (*Meleagris gallopavo*), water buffalos (*Bubalus bubalis*), and humans (Table 3). Most blood sources were chickens (57 of 66 flies, 86%), which were fed on by *S. asakoae*, *S. myanmarense*, *S. khelangense*, *S. aureohirtum*, and *S. striatum* group. *Simulium asakoae* and *S. chumpornense* fed on turkeys, and *S. asakoae* and *S. khelangense* fed on humans. *Simulium luculentum*, *S. striatum* group, and *S. daoense* fed on water buffalos, and *S. chamlongi* fed on humans (Table 3).

## 4. Discussion

Our study demonstrates that an integrated approach is necessary for biodiversity assessments of black flies. Females of the following three species pairs are challenging to identify morphologically: *S. khelangense* and *S. chumpornense*, *S. yvonneae* and *S. laosense*, and *S. asakoae* and *S. myanmarense*. Therefore, only the former species of each pair was recognized morphologically. However, DNA barcoding sequences in our study, compared with those recorded from type localities, indicated the existence of *S. chumpornense*, *S. laosense*, and *S. myanmarense* within *S. khelangense*, *S. yvonneae*, and *S. asakoae*, respectively.

The most abundant taxon in our collections is *S. khelangense*/*S. chumpornense*, representing 58% of all collections. The adults, however, are restricted to areas near large rivers, particularly the Mekong, in agreement with the known habitat of the immature stages, which have thus far been found only in the Mekong River [35]. These species have been recorded in Thailand and Laos [8]. The immature stages of *S. chumpornense* inhabit diverse stream habitats and are geographically widespread in Thailand [35], but in Laos they are found only near the Mekong River [22]. The elevation ranges of the preimaginal habitats in Thailand and Laos indicate that *S. chumpornense* and *S. khelangense* are found below 500 m asl. Other species found only at low (<500 m asl) elevations include *S. yvonneae* and *S. laosense*. Ecological information for their immature stages is limited to data from the type localities in Vietnam [28] and Laos [21], respectively. Both species are found in low-elevation streams (<200 m asl) [21,28], agreeing with the distributions of the adults in our study.

The second most abundant black fly is *S. asakoae*/*S. myanmarense*, representing 38% of the total collections. This taxon is not only relatively abundant, but also geographically widespread at elevations from 174 to 1219 m asl. *Simulium asakoae* is a geographically widespread species, being recorded in Malaysia, Myanmar, China, Thailand, and Vietnam, while *S. myanmarense* is only found in Myanmar and Thailand [8]. The distribution pattern of *S. asakoae* in Laos agrees with that reported for Thailand, where this species could be found from 400 m to 2500 m asl [10,11]. In contrast, adults of *S. asakoae* have been found only at high elevations (>1400 m asl) in Malaysia [12], perhaps because a suitable temperature range can be found only at higher elevations in the more southerly located Malaysia.

*Simulium nodosum* is also found at a wide range of elevations (321–995 m asl), although it was collected from only two sites in Laos. This species has been recorded in many countries: India, Bhutan, Myanmar, China, Thailand, Vietnam, and Laos [8]. The distribution of the adults of *S. nodosum* in Laos agrees with the distribution of the habitats; this species was collected from streams at 200 m to 1100 m asl [22]. *Simulium nodosum* has also been recorded at high elevations in Myanmar (958 m asl) and Vietnam (1439 m asl) [36]. In Thailand, however, it is found only below 800 m asl [10,11]. The *S. striatum* species group is found at a wide elevational range (456–1219 m asl), in agreement with records from Thailand, where the group is found at 400 m to >1000 m asl [11].

Knowledge of the biting habits of black flies can be used to infer the potential of a species to act as a pest or vector. Previously, host blood sources of black flies in Laos were known for only two species, *S. chamlongi* and *S. luculentum*, which attack humans and water buffalos, respectively [16]. *Simulium khelangense* was assumed to feed on chickens because *Leucocytozoon* lineages in chickens were detected in this black fly [17,18,19]. Based on the vertebrate cyt b sequences that we obtained from blood-fed females, the blood sources of black flies in Laos are now known for nine species of black flies. The biting habits of *S. aureohirtum*, *S. daoense*, and *S. myanmarense* are reported here for the first time. *Simulium myanmarense* and *S. aureohirtum* feed on chickens. Although they are in different subgenera (*Gomphostilbia* and *Nevermannia*, respectively), both species have female claws with a large basal tooth adapted for grasping bird feathers [37].

*Simulium daoense* and *S. striatum* group feed on water buffalo and the latter also feeds on chickens, a new host record for this group. Although mammals are the expected hosts of the *S. striatum* group, based on the untoothed female claws [9], birds also can be used as hosts in primarily mammalophilic species [11]. In Thailand, members of the *S. striatum* group feed on water buffalos [11,25]. *Simulium asakoae* in Laos feeds on chickens, humans, and turkeys. The first two hosts were recorded in Thailand [11] and Malaysia [12], whereas turkeys are a new host record. Hosts of *S. khelangense* in Laos are chickens and humans, agreeing with reports in Thailand [11]. The finding that *S. chamlongi* and *S. luculentum* feed on water buffalos and humans, respectively, agrees with previous records in Laos [16]. We found a new host record, turkeys, for *S. chumpornense*. Previously, chickens were the only known host [38].

The hosts of black flies that we identified in Laos are exclusively domestic animals and humans, perhaps, in part, because most collections were made near villages and animal shelters, such as cattle pens, where domestic animals are abundant. The bias toward chickens as hosts (86% of blood-fed flies) might be related to the abundance of *S. asakoae* and *S. khelangense* in our collections, which frequently feed on chickens [11,38]. Collecting adult flies from forested areas will more broadly define the hosts, particularly wild hosts, of black flies in Laos. Opportunities to collect black flies in forested areas, however, are limited by the alarming amount of unexploded ordnance left over from the bombing campaign in the 1970s during the Vietnam War.

Overall, the most abundant species in our study are *S. asakoae* and *S. khelangense*, similar to the findings in Thailand [11]. Because these species attack chickens and are potential vectors of *Leucocytozoon* and *Trypanosoma*, they present a risk to the poultry industry. The adults of other species were found at relatively low abundance. Nonetheless, we were able to provide the first host information for *S. aureohirtum* (chickens) and *S. daoense* (water buffalos). The next logical step in determining the risk factors posed by black flies in Laos is to examine the seasonality of the black flies and which parasites and pathogens they might be transmitting.

## Figures and Tables

**Table 1:** Sampling locations for males, females, and blood-fed females of black flies in Laos, January to March 2025.

Location (Code)	Coordinates	Elevation (m)	Date	Species	Males	Females	Blood-Fed Females	Total
Muang Thaphabat, Bolikhamxai Province (678)	18.231388 N 103.115357 E	172	4 January 2025	*S. khelangense*/ *S. chumpornense*	3	86	-	89
				*S. yvonneae*/ *S. laosense*	-	14	-	14
699			7 February 2025	*S. khelangense*/ *S. chumpornense*	4	503	5	512
Ban Nong Keun, Bolikhamxai Province (679)	18.452807 N 103.406923 E	162	4 January 2025	*S. khelangense*/ *S. chumpornense*	8	312	7	327
				*S. yvonneae*/ *S. laosense*	-	20	-	20
698			9 January 2025	*S. khelangense*/ *S. chumpornense*	-	182	6	188
				*S. yvonneae*/ *S. laosense*	-	3	-	3
700			7 February 2025	*S. khelangense*/ *S. chumpornense*	27	494	4	525
				*S. yvonneae*/ *S. laosense*	-	35	-	35
Borikham, Bolikhamxai Province (680)	18.620325 N 103.737773 E	174	5 January 2025	*S. asakoae*/ *S. myanmarense*	-	58	1	59
				*S. khelangense*/ *S. chumpornense*	-	3	-	3
				*S. yvonneae*/ *S. laosense*	-	1	-	1
701			8 February 2025	*S. khelangense*/ *S. chumpornense*	-	144	4	148
				*S. asakoae*/ *S. myanmarense*	-	87	3	90
				*S. ynonneae*/ *S. laosense*	-	3	-	3
Thathom, Xaisomboun Province (681)	19.037533 N 103.407373 E	321	5 January 2025	*S. asakoae*/ *S. myanmarense*	-	51	1	52
697			8 January 2025	*S. asakoae*/ *S. myanmarense*	-	357	4	361
				*S. yvonneae*/ *S. laosense*	-	1	-	1
712			10 February 2025	*S. asakoae*/ *S. myanmarense*	4	247	1	252
				*S. nodosum*	-	15	-	15
Muang Khoune (1), Xiang Khouang Province (682)	19.228120 N 103.365560 E	995	5 January 2025	*S. asakoae*/ *S. myanmarense*	26	148	8	182
				*S. striatum* group	-	9	-	9
696			8 January 2025	*S. asakoae*/ *S. myanmarense*	4	168	4	176
				*S.* *chamlongi*	-	-	1	1
				*S. striatum* group	-	2	-	2
				*S. nodosum*	-	2	-	2
703			8 February 2025	*S. asakoae*/ *S. myanmarense*	-	57	1	58
				*S. striatum* group	-	2	-	2
Muang Khoune (2), Xiang Khouang Province (683)	19.263842 N 103.361737 E	1045	5 January 2025	*S. asakoae*/ *S. myanmarense*	-	35	-	35
Muang Khoune (3), Xiang Khouang Province (684)	19.374480 N 103.246903 E	1146	5 January 2025	*S. asakoae*/ *S. myanmarense*	-	8	-	8
Muang Kham (1), Xiang Khouang Province (686)	19.637827 N 103.473968 E	756	6 January 2025	*S. asakoae*/ *S. myanmarense*	-	26	3	29
				*S.* *luculentum*	-	-	1	1
				*S. striatum* group	4	9	-	13
695			8 January 2025	*S. asakoae*/ *S. myanmarense*	-	37	2	39
				*S. striatum* group	-	1	3	4
				*S.* *daoense*	-	-	1	1
708			9 February 2025	*S.* *aureohirtum*	7	6	2	15
Vieng Xai, Huaphan Province (690)	20.470123 N 104.143545 E	848	7 January 2025	*S. asakoae*/ *S. myanmarense*	-	6	-	6
Ban Mueang Nga, Vieng Xai, Huaphan Province (691)	20.431587 N 104.178603 E	835	7 January 2025	*S. asakoae*/ *S. myanmarense*	-	93	5	98
Tad Seleuy waterfall, Sam Neua, Huaphan Province (693)	20.231111 N 104.005194 E	1219	7 January 2025	*S. asakoae*/ *S. myanmarense*	-	293	-	293
				*S. striatum* group	-	1	-	1
Ban Nator, Houamuang, Huaphan Province (694)	20.158222 N 103.888923 E	1199	7 January 2025	*S. asakoae*/ *S. myanmarense*	-	8	1	9
Ban Thasi, Muaung Thathom, Nammang River, Xaisomboun Province, (702)	18.847473 N 103.811762 E	209	8 February 2025	*S. asakoae*/ *S. myanmarense*	-	1	0	1
				*S.* *khelangense*	-	1	0	1
Ban Kang Yao, Muang Phonsavan, Xiangkhoang Province (709–710)	19.376930 N 103.168240 E	1119	9 February 2025	*S. asakoae*/ *S. myanmarense*	-	2	0	2
Sokbounma hotel, Muang Pakxane, Borikhamxai Province (713)	18.392865 N 103.641953 E	159	11 February 2025	*S. khelangense*/ *S. chumpornense*	-	337	-	337
Muang Vangvieng, Nam Xong River, Vientiane Province (715)	19.018830 N 102.446027 E	250	21 March 2025	*S. khelangense*/ *S. chumpornense*	-	1	-	1
Ban Pha Hom, Muang Vangvieng, Vientiane Province (716)	19.125217 N 102.344787 E	478	21 March 2025	*S. striatum* group	-	2	-	2
Ban Nong, Muang Kasi, Vientiane Province (717)	19.125457 N 102.249120 E	456	21 March 2025	*S. khelangense*/ *S. chumpornense*	-	3	-	3
				*S. asakoae*/ *S. myanmarense*	-	38	-	38
				*S. striatum* group	-	2	-	2
Ban Lao Kham (1), Muang Feuang, Vientiane Province (726)	18.652842 N 102.110338 E	227	22 March 2025	*S. khelangense*/ *S. chumpornense*	2	268	2	272
Ban Na Keo, Muang Thoulakhom, Vientiane Province (728)	18.341101 N 102.643600 E	176	23 March 2025	*S. khelangense*/ *S. chumpornense*	-	294	24	318

**Table 2:** Males, females, blood-fed females, and elevation ranges of adult black flies (*Simulium* species) from Laos, January–March 2025.

Subgenus Species	Males	Females	Blood-Fed Females	Total	% OC	% OL	Elevation Range (m)
subgenus *Gomphostilbia*							
*S. asakoae*/ *S. myanmarense*	34	1720	34	1788	63.3	65.0	174–1219
*S. khelangense*/ *S. chumpornense*	44	2628	52	2724	43.3	45.0	159–456
*S. yvonneae*/*S. laosense*	0	77	0	77	23.3	20.0	162–321
subgenus *Nevermannia*							
*S.* *aureohirtum*	7	6	2	15	3.3	5.0	756
subgenus *Simulium*							
*S.* *chamlongi*	0	0	1	1	3.3	5.0	995
*S.* *daoense*	0	0	1	1	3.3	5.0	756
*S.* *luculentum*	0	0	1	1	3.3	5.0	756
*S. nodosum*	0	17	0	17	6.7	10.0	321–995
*S. striatum* group	4	28	3	35	23.3	25.0	456–1219
Total	89	4476	93	4658			

Note: OC, occurrence in collection; OL, occurrence in location.

**Table 3:** Molecular identification of blood hosts of black flies in Laos, based on mitochondrial cyt b gene sequences.

Subgenus/Species		Blood Hosts	
Morphological identification (*n*)	Molecular (total blood engorged/successful identification)	Host species	*n*
subgenus *Gomphostilbia*			
*S. asakoae* (30)	*S. asakoae* (27/14)	Chicken (*Gallus gallus*)	11
		Turkey (*Meleagris gallopavo*)	2
		Human (*Homo sapiens*)	1
	*S. myanmarense* (3/2)	Chicken (*Gallus gallus*)	2
*S. khelangense* (52)	*S. khelangense* (48/47)	Chicken (*Gallus gallus*)	42
		Human (*Homo sapiens*)	1
	*S. chumpornense* (4/1)	Turkey (*Meleagris gallopavo*)	1
subgenus *Nevermannia*			
*S. aureohirtum* (2)	*S. aureohirtum* (2/1)	Chicken (*Gallus gallus*)	1
subgenus *Simulium*			
*S. luculentum* (1)	*S. luculentum* (1)	Water buffalo (*Bubalus bubalis*)	1
*S. chamlongi* (1)	*S. chamlongi* (1)	Human (*Homo sapiens*)	1
*S. striatum* group (2)	*S. striatum* group (2)	Water buffalo (*Bubalus bubalis*)	1
		Chicken (*Gallus gallus*)	1
*S. daoense* (1)	*S. daoense* (1)	Water buffalo (*Bubalus bubalis*)	1

## Data Availability

The sequences have been deposited into the NCBI GenBank under the accession numbers PX314873–PX314957 and PX349369–PX349386. All other data and materials supporting this article are available from the corresponding author, P. P., upon request.

## References

[1] Adler P.H., McCreadie J.W., Mullen G., Durden L.A. (2019). Black flies (Simuliidae). Medical and Veterinary Entomology.

[2] WHO Onchocerciasis. https://www.who.int/news-room/fact-sheets/detail/onchocerciasis.

[3] Takaoka H., Fukuda M., Otsuka Y., Aoki C., Uni S., Bain O. (2012). Blackfly vectors of zoonotic onchocerciasis in Japan. Med. Vet. Entomol..

[4] Valkiūnas G. (2005). Avian Malaria Parasites and Other Haemosporidia.

[5] Reisen W.K. (2010). Landscape epidemiology of vector-borne diseases. Annu. Rev. Entomol..

[6] Martínez-de la Puente J., Figuerola J., Soriguer R. (2015). Fur or feather? Feeding preferences of species of *Culicoides* biting midges in Europe. Trends Parasitol..

[7] Kitron U. (1998). Landscape ecology and epidemiology of vector-borne diseases: Tools for spatial analysis. J. Med. Entomol..

[8] Adler P.H. (2025). World Blackflies (Diptera: Simuliidae): A Comprehensive Revision of the Taxonomic and Geographical Inventory. http://biomia.sites.clemson.edu/pdfs/blackflyinventory.pdf.

[9] Takaoka H. (2024). The Black Flies of Subtropical and Tropical Asia: Taxonomy and Biology.

[10] Srisuka W., Sulin C., Aupalee K., Phankaen T., Taai K., Thongsahuan S., Saeung A., Takaoka H. (2021). Community structure, biodiversity and spatiotemporal distribution of the black flies (Diptera: Simuliidae) using malaise traps on the highest mountain in Thailand. Insects.

[11] Gomontean B., Jumpato W., Wongpakam K., Tangkawanit U., Wannasingha W., Thanee I., Ya’cob Z., Pramual P. (2024). Diversity, distribution and host blood meal analysis of adult black flies (Diptera: Simuliidae) from Thailand. Insects.

[12] Izwan-Anas N., Halim M.R.A., Low V.L., Adler P.H., Ya’cob Z. (2024). Wild-caught adult black flies (Diptera: Simuliidae) from various ecological landscapes in Malaysia. Acta Trop..

[13] Mukherjee A., Kar O., Mukherjee K., Mukherjee B., Naskar A., Banerjee D. (2025). Molecular identification of Onchocerciasis vectors (Diptera: Simuliidae) from the central Himalayan landscape of India: A DNA barcode approach. Vector Borne Zoonotic Dis..

[14] Pramual P., Tangkawanit U., Kunprom C., Vaisusuk K., Chatan W., Wongpakam K., Thongboonma S. (2020). Seasonal population dynamics and a role as natural vector of *Leucocytozoon* of black fly, *Simulium chumpornense* Takaoka & Kuvangkadilok. Acta Trop..

[15] Pramual P., Petney T.N., Saijuntha W., Mehlhorn H. (2021). Black fly diversity and impacts on human welfare in Southeast Asia. Biodiversity of Southeast Asian Parasites and Vectors Causing Human Disease.

[16] Adler P.H., Gomontean B., Jumpato W., Mintara R., Namtaku S., Thanee I., Wannasingha W., Wongpakam K., Jaroenchaiwattanachote C., Inkhavilay K. (2025). An integrated analysis of mammalophilic blackflies in the *Simulium variegatum* group (Diptera: Simuliidae) in Laos. Med. Vet. Entomol..

[17] Jumpato W., Tangkawanit U., Wongpakam K., Pramual P. (2019). Molecular detection of *Leucocytozoon* (Apicomplexa: Haemosporida) in black flies (Diptera: Simuliidae) from Thailand. Acta Trop..

[18] Jumpato W., Wannasingha W., Jaroenchaiwattanachote C., Mintara R., Wongpakam K., Adler P.H., Pramual P. (2024). Diversity and prevalence of *Leucocytozoon* in black flies (Diptera: Simuliidae) of Thailand. Parasit. Vectors.

[19] Gomontean B., Jumpato W., Namtaku S., Wannasingha W., Wongpakam K., Thanee I., Inkhavilay K., Malavong B., Pramual P. (2025). Black fly diversity and molecular detection of blood parasites in *Simulium khelangense* (Diptera, Simuliidae) from Laos. J. Med. Entomol..

[20] Pramual P., Nanork P. (2012). Phylogenetic analysis based on multiple gene sequences revealing cryptic biodiversity in *Simulium multistriatum* group (Diptera: Simuliidae) in Thailand. Entomol. Sci..

[21] Takaoka H., Srisuka W., Saeung A., Maleewong W., Low V.L. (2017). A new black fly species of *Simulium* (*Gomphostilbia*) (Diptera: Simuliidae) from Laos. J. Med. Entomol..

[22] Thanee I., Gomontean B., Jumpato W., Namtaku S., Wongpakam K., Wannasingha W., Inkhavilay K., Malavong B., Pramual P. (2014). Genetic characterization and breeding habitats of black fly (Diptera, Simuliidae) vector species in Laos. Diversity.

[23] Adler P.H., Vlasov S., Huang Y.T., Hadi U.K., Inkhavilay K., Malavong B., Topolenko V., Gomontean B., Jumpato W., Mintara R. (2025). Rare chromosomal uniformity in black flies of the *Simulium striatum* species group (Diptera: Simuliidae). Insects.

[24] Adler P.H., Wannasingha W., Gomontean B., Jumpato W., Mintara R., Namtaku S., Thanee I., Wongpakam K., Jaroenchaiwattanachote C., Inkhavilay K. (2025). Laos, a New Frontier for Investigating Black Flies (Diptera: Simuliidae). J. Med. Entomol..

[25] Takaoka H., Choochote W., Aoki C., Fukuda M., Bain O. (2003). Black flies (Diptera: Simuliidae) attracted to humans and water buffalos and natural infections with filarial larvae, probably *Onchocerca* sp., in northern Thailand. Parasite.

[26] Fukuda M., Choochote W., Bain O., Aoki C., Takaoka H. (2003). Natural infections with filarial larvae in two species of black flies (Diptera: Simuliidae) in northern Thailand. Jpn. J. Trop. Med. Hyg..

[27] Takaoka H., Srisuka W., Saeung A. (2019). Checklist and keys for the black flies (Diptera: Simuliidae) in Thailand. Med. Entomol. Zool..

[28] Takaoka H., Sofian-Azirun M., Ya’Cob Z., Chen C.D., Lau K.W., Low V.L., Da Pham X., Adler P.H. (2017). The black flies (Diptera: Simuliidae) of Vietnam. Zootaxa.

[29] Folmer O., Black M., Hoeh W., Lutz R., Vrijenhoek R. (1994). DNA primers for amplification of mitochondrial cytochrome c oxidase subunit I from diverse metazoan invertebrates. Mol. Mar. Biol. Biotechnol..

[30] Tangkawanit U., Wongpakam K., Pramual P. (2018). A new black fly (Diptera: Simuliidae) species of the subgenus *Asiosimulium* Takaoka Choochote from Thailand. Zootaxa.

[31] Kocher T.D., Thomas W.K., Meyer A., Edwards S.V., Pääbo S., Villablanca F.X., Wilson A.C. (1989). Dynamics of mitochondrial DNA evolution in animals: Amplification and sequencing with conserved primers. Proc. Nat. Acad. Sci. USA.

[32] Malmqvist B., Strasevicius D., Hellgren O., Adler P.H., Bensch S. (2004). Vertebrate host specificity of wild–caught blackflies revealed by mitochondrial DNA in blood. Proc. R. Soc. Lond. B.

[33] Kumar S., Stecher G., Li M., Knyaz C., Tamura K. (2018). MEGA X: Molecular evolutionary genetics analysis across computing platforms. Mol. Biol. Evol..

[34] Putt Q.Y., Ya’cob Z., Adler P.H., Chen C.D., Hew Y.X., Izwan-Anas N., Lau K.W., Sofian-Azirun M., Pham X.D., Takaoka H. (2023). From bites to barcodes: Uncovering the hidden diversity of black flies (Diptera: Simuliidae) in Vietnam. Parasit. Vectors.

[35] Thanee I., Jumpato W., Jaroenchaiwattanachote C., Gomontean B., Wannasingha W., Namtaku S., Adler P.H., Pramual P. (2024). Discovery of the larvae and pupae of the black fly *Simulium* (*Gomphostilbia*) *khelangense* and breeding habitats of potential pest species of the *S*.(*G*.) *chumpornense* subgroup (Simuliidae). Insects.

[36] Low V.L., Adler P.H., Sofian-Azirun M., Srisuka W., Saeung A., Huang Y.T., Hadi U.K., Da Pham X., Takaoka H. (2015). Tests of conspecificity for allopatric vectors: *Simulium nodosum* and *Simulium shirakii* (Diptera: Simuliidae) in Asia. Parasit. Vectors.

[37] Adler P.H., Currie D.C., Wood D.M. (2004). The Black Flies (Simuliidae) of North America.

[38] Pramual P., Thaijarern J., Tangkawanit U., Wongpakam K. (2020). Molecular identification of blood meal sources in black flies (Diptera: Simuliidae) suspected as *Leucocytozoon* vectors. Acta Trop..

